# Vitamin D (25OHD) Serum Seasonality in the United States

**DOI:** 10.1371/journal.pone.0065785

**Published:** 2013-06-21

**Authors:** Amy K. Kasahara, Ravinder J. Singh, Andrew Noymer

**Affiliations:** 1 Program in Public Health, University of California Irvine, Irvine, California, United States of America; 2 Division of Clinical Biochemistry and Immunology, Mayo Clinic, Rochester, Minnesota, United States of America; 3 Department of Population Health and Disease Prevention, University of California Irvine, Irvine, California,United States of America; Aga Khan University, Pakistan

## Abstract

**Background:**

Vitamin D is an important micronutrient for health. Hypovitaminosis D is thought to play a role in the seasonality of a number of diseases and adverse health conditions. To refine hypotheses about the links between vitamin D and seasonal diseases, good estimates of the cyclicality of serum vitamin D are necessary.

**Objectives:**

The objective of this study is to describe quantitatively the cyclicality of 25-hydroxyvitamin D (25OHD) in the United States. We provide a statistical analysis with weekly time resolution, in comparison to the quarterly (winter/spring/summer/fall) estimates already in the literature.

**Methods:**

We analyzed time series data on 25OHD, spanning 287 consecutive weeks. The pooled data set comes from 3.44 million serum samples from the United States. We statistically analyzed the proportion of sera that were vitamin D sufficient, defined as 25OHD 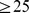 ng/mL, as a function of date.

**Results:**

In the United States, serum 25OHD follows a lagged pattern relative to the astronomical seasons, peaking in late summer (August) and troughing in late winter (February). Airmass, which is a function of solar altitude, fits the 25OHD data very well when lagged by 8 weeks.

**Conclusions:**

Serum vitamin D levels can be modeled as a function of date, working through a double-log transformation of minimal solar airmass (easily calculated from solar altitude, retrievable from an online solar altitude/azimuth table).

## Introduction

There are many hypotheses about the role of hypovitaminosis D in the seasonality of infectious disease [1]–[5], chronic obstructive pulmonary disease [6], [7], cancer [8]–[11], fractures [12], healthy pregnancy [13], and other maladies [14]–[16]. The principal mechanism for acquisition of vitamin D is cutaneous UVB photosynthesis of vitamin D_3_ (cholecalciferol) from 7-dehydrocholesterol [17]. Dietary intake from supplements, fortified foods (especially dairy), and foods (e.g., egg yolks, oily fish) that naturally contain vitamin D also affect vitamin D levels [18], [19].

Despite the potentially enormous influence of vitamin D on health, there is a paucity of population-based data on the seasonality of serum vitamin D levels [20]. This paper provides estimates of the temperate boreal seasonality of serum 25-hydroxyvitamin D (hereinafter 25OHD), which may be extrapolated to other studies. The intent is to be descriptive: the causal aspect of serum vitamin D cyclicality is already well understood in terms of skin exposure to solar ultraviolet (UVB) radiation [21]. However, the detailed empirics of when serum 25OHD levels peak and trough during the year has not heretofore been well-characterized with large sample size studies.

## Materials and Methods

### Ethics statement

The original sera collection and 25OHD analysis was approved by the IRB of the Mayo Clinic, Rochester, Minnesota, USA. The secondary analysis for seasonality, presented herein, uses only nonidentifiable aggregate data.

### 25OHD serum data

We analyzed pooled data on 25OHD serum levels from 3,440,710 individual samples sent to the Mayo Clinic (Rochester, Minnesota, USA) from all over the United States. Serum collection ranged from July 2006 to December 2011, dated with weekly precision. The large sample size is one of the strengths of this study, because it provides reliable estimates of the population prevalence of vitamin D sufficiency, defined herein as serum 25OHD 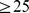 ng/mL (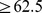 nmol/L). The explanandum of our study, and the dependent variable in the statistical models we estimated, is not the mean 25OHD level (which is not calculable from our data), but the proportion of sera that are vitamin D (25OHD) sufficient. In addition to the analysis of sufficient (25OHD 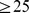 ng/mL, vs. 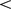
25), we present a disaggregated analysis according to four categories of 25OHD levels (all quantities in ng/mL): (i) 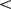
10; (ii) 10–24; (iii) 25–80; (iv) 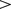
80. Due to the binned nature of the data as they are available, there are no more detailed categories than these.

Serum 25OHD is a difficult analyte [22], [23]. Our data come from techniques designed to overcome these measurement difficulties as much as possible [24]. The sample includes all sera sent to the endocrinology lab at the Mayo Clinic and is not limited to patients with suspected hyper- or hypovitaminosis D. The sample is representative of the population of the United States interacting with health care. The data are aggregated and do not permit analysis by age, sex, geography, or other co-variates. However, with almost 3.5 million sera, this is by far the largest sample-size study of which we are aware to examine the seasonality of vitamin D levels. Prior studies have assayed far fewer samples [25]–[28].

### Solar radiation data

Since 25OHD production is a function of skin exposure to ultraviolet light (UVB), there is a strong *a priori* expectation that incident solar radiation will be highly correlated with serum 25OHD levels. We obtained the weekly maximum solar altitude (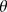) for Rochester, Minnesota, USA [29]. Airmass measures how much atmosphere the light from an astronomical object must pass through before reaching the earth's surface. The minimal solar airmass is approximated according to: airmass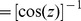, where 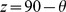 is the minimal zenith angle of the sun in degrees [30]. Airmass, which is unitless, is an inverse relation with incident solar radiation: it is lowest (1.0) when the sun is directly overhead, as occurs in the tropics, and is highest (approaching 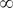) when the sun is rising or setting. We expect greater 25OHD levels when the daily minimum solar airmass is small (i.e., the sun is high above the horizon). Rather than solar altitude (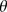) or calendar date, we analyzed the data in terms of airmass since it is more closely related to the mechanisms of vitamin D synthesis (i.e., incident solar UVB radiation).

It has long been recognized that weather as well as season can affect vitamin D levels [31]. Incident solar UV radiation is a function of cloud cover as well as airmass. Moreover, other localized atmospheric factors, such as ozone, can affect ground-level UVB [32], [33]. Single-site studies have measured incident solar radiation with a UV photometer [34]. However, because our data are pooled from all over the United States, weather data for a specific location are not helpful for modelling our 25OHD data set. To test whether general, large-scale, atmospheric patterns have any association with 25OHD levels, we obtained data on the Oceanic Niño Index (ONI) from the US National Weather Service [35]. The ONI is an ocean temperature-based index of the El Niño/La Niña climatic pattern, which affects weather on a nearly continental scale, especially in the western United States.

Because the Mayo Clinic is a reference laboratory, our data include serum samples from all over the United States. The use of a specific city (in this case, Rochester, Minnesota, where the Mayo Clinic is located) as the source of airmass data is applicable because the variation over time of minimal airmass of a given city is proportional to an average of the United States. In other words, a linear combination (i.e., a multiplier and an offset) of log-log airmass for Rochester captures the weekly variation of log-log airmass for a weighted average of all cities in the United States.

**Figure 1:**
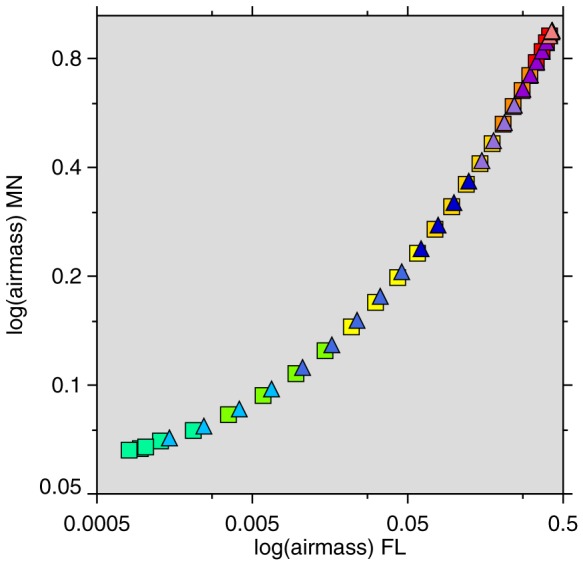
Weekly log(airmass) Rochester, Minnesota versus log(airmass) Miami, Florida (on log scale, thus double-log). Colors signify month. January–June, squares; July–December, triangles.

To illustrate this, we made an artificially-exaggerated (i.e., worst-case-scenario) comparison: figure 1 plots log(log(airmass)) for Rochester, Minnesota versus the same quantity for Miami, Florida, the southernmost metropolitan area in the contiguous United States. There is a very tight fit between Rochester and Miami: 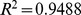 for a straight-line model in figure 1 (a quadratic improves the fit to 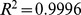, but the 25OHD models do not fit better with a quadratic specification [compare table 1, model 1 and model 2, discussed below]).

**Table 1:** Regression table; see text for complete discussion.

	Proportion of samples 25OHD≥25 ng/mL				
	(1)	(2)	(3)	(4)	(5)
log(log(airmass))	−0.0556**	−0.0567**	−0.0557**	0.0104	−0.0556**
	(−32.6)	(−8.22)	(−34.2)	(0.21)	(−32.6)
time	0.656**	0.656**	0.835**	0.832**	0.656**
	(7.43)	(7.42)	(9.23)	(9.17)	(7.43)
time^2^	−0.00648**	−0.00648**	−0.00829**	−0.00826**	−0.00648**
	(−7.29)	(−7.27)	(−9.08)	(−9.03)	(−7.29)
[log(log(airmass))]^2^		−0.000395			
		(−0.16)			
ONI			−0.00990**	−0.0101**	
			(−5.39)	(−5.44)	
Constant	−15.93**	−15.93**	−20.36**	−20.22**	−15.93**
	(−7.29)	(−7.28)	(−9.08)	(−8.99)	(−7.29)
cos ( 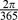 ×time)				−0.0622	
				(−1.37)	
sin ( 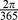 ×time)				−0.0588	
				(−1.30)	
cos ( 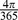 ×time)				0.000848	
				(0.40)	
sin ( 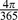 ×time)				0.00767	
				(1.25)	
Observations	287	287	287	287	287
*R^2^*	0.82	0.82	0.84	0.84	0.82

*t* statistics in parentheses.

** *p*<0.0001,

* *p*<0.001.

Model (3) is the final descriptive model, performing better than models (1) and (2). Model (4) shows that seasonal (sin, cos) terms provide no improvement upon model (3), and model (5) is the out-of-sample prediction model for periods in which the Oceanic Niño Index (ONI) is not available. Time is measured in days since 1 January 1960.

## Results

**Figure 2:**
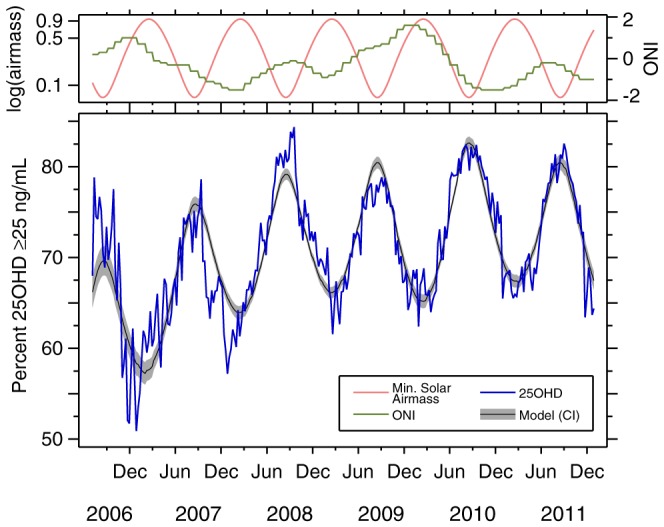
Time series plot of 25OHD data and covariates. Top panel plots model covariates. Left axis: logarithm of minimum solar airmass, 8-week lag (light red, on log scale, thus double-log); right axis: Oceanic Niño Index (green). Bottom panel plots proportion of serum samples testing 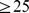 ng/mL (blue), and model (black), 95% confidence bounds (gray), from model 3 in table 1.

The data are shown in figure 2. The bottom panel plots the raw data in blue; this is the percent of serum samples testing 25OHD 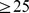 ng/mL. These data are superimposed on the model results (black) with confidence bounds (gray); the modelling strategy is discussed below. The top panel of figure 2 plots the covariates used in the model: log(log(airmass)) (left vertical axis, in light red), which has a sinusoid-like shape; and the Oceanic Niño Index (ONI) (right vertical axis, in green). Table 1 presents five regression models, fitting the proportion 25OHD 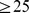 ng/mL as a function of the listed covariates, using ordinary least squares regression on the 287 weekly observations. Each observation is weighted by the number of serum samples on which it is based (the number of samples, by season, is given in table 2; the average is 11,988 serum samples per week, with 2006 having fewer due to the initiation of the study).

**Table 2:** Season-by-season summary of 25OHD peaks and troughs.

I	II	III	IV	V	VI	VII	VIII	IX
			Observed			Model		O–M date
		Number of samples	Min/Max		Δ	Min/Max		(weeks)
Season			Value	Date		Value	Date	
S/S	2006	3,018	0.7881	7/12	n/a	0.6963	8/23	−6
F/W	2006–07	79,897	0.5088	12/27	0.279	0.5724	1/31	−5
S/S	2007	214,830	0.7861	9/12	0.277	0.7590	8/22	3
F/W	2007–08	260,304	0.5719	12/26	0.214	0.6393	2/13	−7
S/S	2008	353,859	0.8437	9/17	0.272	0.7917	8/13	5
F/W	2008–09	419,315	0.6155	2/18	0.228	0.6611	2/11	1
S/S	2009	472,216	0.7883	9/02	0.173	0.8045	8/19	2
F/W	2009–10	449,799	0.6240	1/27	0.164	0.6517	2/17	−3
S/S	2010	418,075	0.8239	9/15	0.200	0.8261	8/18	4
F/W	2010–11	311,483	0.6548	12/29	0.169	0.6725	3/02	−9
S/S	2011	302,262	0.8254	8/31	0.171	0.8045	8/17	2
F/W	2011–12	155,652	0.6341	11/23	0.191	n/a	n/a	n/a
Total		3,440,710						

Half-year periods (columns I and II) are defined equinox-to-equinox (S/S, “spring/summer”; F/W, “fall/winter”). Column III gives the total number of serum samples in each period. Column IV gives the proportion of samples testing 25OHD 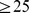 ng/mL for the minimum (maximum) week in the half-year period, and column V gives the corresponding mid-week date (month/day). Column VI, 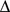, gives the absolute value of the peak-to-trough difference in column IV. Columns VII and VIII give the model prediction and mid-week date of the minimum (maximum) 25OHD proportion, based on model 3 of table 1. Column IX gives the difference in timing between columns V and VIII. The fall/winter 2011–12 model-predicted minimum falls beyond the observed data, so no comparison is made.

We know from first principles that solar radiation is closely related to serum 25OHD levels. However, the functional form of the relationship between airmass and 25OHD serum levels is unknown. Given this ignorance of the exact nature of the relationship, we wish to find some relatively basic transformation which fits the data, as in the ‘ladder of powers’ approach [36]. Model 1 is a basic model, fitting log(log(airmass)) and a quadratic time trend. The double-log specification provided a better fit than untransformed airmass, or single-log, quadratic, or square root transformations. To maximize model fit, airmass was lagged by 8 weeks in all models; the lag was chosen from trial of all possible lags, 0–24 weeks.

Model 2 (table 1) tests a quadratic specification of log(log(airmass)) but it is not significant. Model 3 includes the Oceanic Niño Index, which is highly statistically significant and improves the model fit to 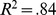. This shows that the ONI is correlated with the proportion of sera which test 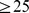 ng/mL, even in a model also including airmass. Because of the cyclicality in the 25OHD levels, model 4 tests seasonal terms (linear combination of sine and cosine) with annual (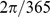) and six-month (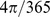) components [37]. This approach is similar to that of some prior work on vitamin D [27]. The seasonal terms are neither jointly statistically significant (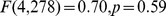), nor individually (see table 1), and do not improve the goodness-of-fit (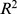) of the model. The log-log specification of airmass, which is itself sinusoidal-like (figure 2), captures the observed periodicity of the data well enough that the model is not improved by addition of these seasonal terms. Thus, model 3 is our final descriptive model.

Table 2 summarizes the seasonal findings, presenting minima and maxima of the proportion of samples 25OHD 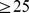 ng/mL from the raw data, and as predicted by model 3. The model fits a sinusoid-like waveform, and therefore a unique maximum (minimum) in every spring/summer (fall/winter). As figure 2 demonstrates, the data exhibit random week-to-week variation, explaining why the observed minus model timing (O
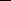
M date, column IX) is never dead-on. As would be expected given that the seasonal (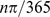) terms in model 4 (table 1) were not significant, there is no consistent pattern in the O
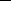
M (column IX of table 2). Both the peaks (spring/summer) and troughs (fall/winter) experience positive and negative O
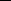
M dates, and the RMS deviation of peaks (3.96 weeks) and troughs (5.74 weeks) differs by less than two weeks. Column III gives the sample size, which accounts for the variation in the width of the confidence band in figure 2 (more samples, narrower band).

The time-trend (i.e., as opposed to seasonal) component of the 25OHD levels is increasing; the trend is modest but statistically significant (table 1). This may be due to the increasing use of multivitamin supplementation in the US [38], [39]. Column VI of table 2 shows that the peak-to-trough amplitude of the raw data in figure 2 is decreasing over time. Two important caveats to this observation are (i) that there are only 11 such amplitudes, so the sample size is too small to make a sweeping generalization, and (ii) the first two observed amplitudes, which are also the largest and second-largest, are based on by far the smallest number of samples, and are therefore more subject to random variation. However, the decrease in amplitude of 25OHD levels over time is also consistent with observed increases in (multi)vitamin supplement use. In other contexts, vitamin D supplementation has been implicated in the flattening of seasonal patters [40].

Vitamin D (25OHD) serum levels follow the long-understood pattern of the ‘lag of the seasons’ (originally from the context of air temperature being out of phase with astronomical seasons [41]). This is seen with the 8-week lag of airmass giving the best model fit (as discussed above), and, more directly, that the 25OHD models peak (trough) in August (February). This may reflect a gradual bioaccumulation of vitamin D during the months in which there is significant photosynthesis (and similarly a gradual diminution of reserves in the months when the sun is low). Dietary intake of vitamin D, especially through fortified food and/or multivitamin supplements [42] is one factor that can explain the relatively low prevalence of hypovitaminosis D (with the smallest proportion of samples testing 25OHD 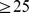 ng/mL still being above 50% [table 2]). Our results on proportion of the population with vitamin D sufficiency are not directly comparable to studies of mean 25OHD levels, but are in broad agreement with other work that has looked at seasonal sufficiency [27].

The out-of-sample prediction equation, from model (5), which necessarily omits the Oceanic Niño Index (ONI) because it is not available in some past nor any future periods, is:
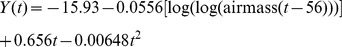
where 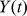 is the proportion of samples testing 25OHD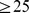 ng/mL at time 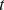, measured in days since 1 January 1960; airmass is lagged by 56 days (8 weeks) as discussed above. Airmass for any location at any date can be calculated using solar altitude data from astronomical tables [29].

**Figure 3:**
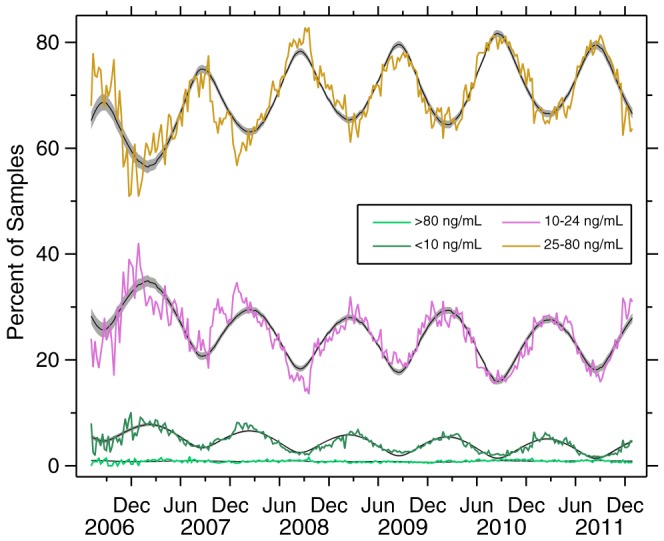
Time series plot of 25OHD data, four categories. Each color represents one 25OHD category, as indicated. Models (black) and 95% confidence bounds (gray) from models in table 3 are also shown. All four series total 100%.

Figure 3 presents the results of the more disaggregated analysis. The categories, from top to bottom of the graph (most to least prevalent), are: 25–80, 10–24, 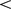
10, 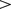
80 ng/mL. It is important to note that all four categories are constrained to sum to 100%, which is why the two principal categories (25–80 and 10–24) are perfectly out-of-phase with each other; when the proportion 25–80 is highest, the proportion 10–24 lowest, and vice versa. The proportion of the samples with 25OHD 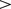
80 is very low (almost negligible, and zero in some weeks). Note that our principal analysis (figure 2 and tables 1 and 2) consists of the sum of the 25–80 and 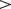
80 categories from figure 3.

Table 3 presents a statistical analysis of the data in figure 3. The models plotted in figure 3, above which the raw data are superimposed, come from this table. The first model in table 3 is a recapitulation of model (3) from table 1, and is provided for comparison. The remaining four models use the same model specification, but with the indicated categories as the dependent variable. The lag producing the best fit was identified separately for each category, but was always 8 weeks, except for 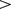
80, which was 3 weeks.

**Table 3:** Models of categories of 25OHD serum data.

	Proportion of samples in each 25OHD category (ng/mL)				
	(≥25)	(< 10)	(10–24)	(25–80)	(> 80)
log(log(airmass))	−0.0557**	0.0140**	0.0417**	−0.0553**	−0.000522**
	(−34.2)	(36.5)	(29.9)	(−35.8)	(−4.34)
time	0.835**	−0.147**	−0.688**	0.850**	−0.0151
	(9.23)	(−6.89)	(−8.88)	(9.88)	(−2.28)
time^2^	−0.00829**	0.00143**	0.00687**	−0.00844**	0.000154
	(−9.08)	(6.64)	(8.78)	(−9.73)	(2.29)
ONI	−0.00990**	0.000611	0.00929**	−0.00940**	−0.000534*
	(−5.39)	(1.41)	(5.90)	(−5.38)	(−3.94)
Constant	−20.36**	3.820**	17.54**	−20.74**	0.381
	(−9.08)	(7.24)	(9.13)	(−9.73)	(2.31)
Observations	287	287	287	287	287
R^2^	0.84	0.87	0.79	0.85	0.15 lag
(weeks)	8	8	8	8	3

*t* statistics in parentheses.

** *p*<0.0001,

* *p*<0.001.

The first column (“
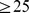
”) is the same as model (3) in table 1. The remaining four columns give regression coefficients for the same model specification, using the category-specific subsets of the data (as indicated in ng/mL) as the dependent variable. The lag of airmass was independently identified for each model, but was always 8 weeks, except for 25OHD 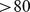 ng/mL, for which the lag was 3 weeks.

## Discussion

In the temperate northern hemisphere, serum 25OHD levels are cyclical, with peaks in August and troughs in February. Thus, like air temperature, vitamin D shows a ‘lag of the seasons’ pattern. Epidemiologists using temporal data to establish association between vitamin D and disease incidence should use this pattern — and not seasonality per se — as a benchmark. For example, a seasonal pattern without lag (i.e., with maxima/minima in late June/December) would not be consistent with an effect of serum 25OHD levels. We find a double-log specification of minimum daily solar airmass to be the best fit to the 25OHD data. This implies diminishing returns to higher solar altitude for serum 25OHD levels. In other words, small week-to-week differences in solar altitude in the summer months, when the sun is already high enough to ensure vitamin D photosynthesis, are less important than same-magnitude differences in late winter when changes occur relative to a lower maximal solar altitude.

If the physiological processes in the putative causal link between hypovitaminosis D and disease involve cascading effect chains, then the impact of serum 25OHD levels on clinical disease may lag even more. For example, if hypovitaminosis D weakens the immune system [43]–[45], this can lead to infection, which can in turn take a week or more to manifest clinically. The use of time series data to establish a statistical association between a disease and vitamin D must take timing into account more precisely than crude winter/summer or 4-seasonal approaches. We provide a prediction equation which may be used for extrapolation to other studies where direct measurement of serum 25OHD is not available.

This study has several limitations. The proportional analysis is necessitated by the data binning, which prevents calculation of mean 25OHD levels. We have no more detailed information than the categories presented herein. However, all the available evidence point to the shape of figure 1 being the same even if it were a graph of mean 25OHD.

In theory, having weather data, especially cloud cover information, would be useful. However, because of the nationally pooled nature of our sample, we are unable to include local weather in our analysis. We believe that our large sample size (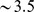 million) design is a major strength, but, if possible, future studies should include local cloud cover data (or direct ambient UVB measurement). Our statistically-significant finding with respect to the Oceanic Niño Index (ONI) implies that local weather data would be even more important. Moreover, the modestly large week-to-week noise on top of the signal in figure 2 (the RMS error of model 3 is .02514) is an indirect indication that weather may play a reasonably important role in vitamin D levels. Use of sunscreen and avoidance of the sun are factors that attenuate vitamin D photosynthesis. Therefore, lack of behavioral information in our data is a source of unobserved heterogeneity.

Another limitation of the sample is the lack of demographic and geographic covariates. Differences in skin pigmentation affect vitamin D photosynthesis, but we have no individually-linked data with which to conduct such an analysis. Rates of vitamin supplementation may also vary by age [40]. The geographic composition of the sample does not change over time, so a potential source of systematic bias — for instance from including more northerly cities in the winter, when there may be concern about hypovitaminosis D — is not an issue in our data set.

The disaggregated analysis (figure 3 and table 3) does not yield many additional insights. This is because all four data series in the disaggregated analysis are constrained to add to 1.0, and the 25OHD 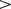
80 ng/mL category is a negligible portion of the population. Nonetheless, it is worth noting that even among the vitamin D sufficient population, 25OHD levels 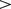
80 ng/mL are rare. What is more, the pattern of this category shows much less seasonal lag (3 weeks, table 3), which is not surprising given the UVB levels necessary for such high levels of vitamin D production. It is also noteworthy that among the samples 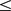
25 ng/mL 25OHD, most are still 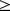
10 ng/mL.

Ordinary least squares regression (OLS) has the potential shortcoming of producing model estimates that need not be true proportions (i.e., 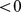 or 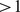). However, as is evident from figure 2, this concern does not arise in this case. Moreover, the point of the statistical models herein is curve-fitting, not modeling to determine causal structure (which is already known to be solar UVB). Thus, since OLS is perfectly well-behaved in this case, the use of models constrained to produce only proportions (such as GLM [46]), is not necessary.

Despite these limitations, our data provide robust estimates of the seasonality of serum 25OHD using the largest-ever sample size for such a study (almost 3.5 million sera). The serum data come from state-of-the-art analytical techniques [24]. Although having demographic covariates would allow refinement of the regression estimates, most studies are interested in whole-population vitamin D levels, and that is what our data represent. In summary, we have no information on any covariates that are not already presented in table 1. Nonetheless, the models we present fit the data very well, with 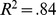 from a model with five independent variables.

In conclusion, we have quantitatively characterized the seasonality of serum vitamin D levels in the United States. The functional form of our statistical model, relating vitamin D seasonality to astronomical seasons through a double-log transformation of solar minimal airmass, is novel. In terms of phase (cycle), these results ought to be applicable to all temperate northern hemisphere populations. With the appropriate 6-month shift, our results can be extended to the temperate southern hemisphere. However, because of differences in diet, supplement use, latitude, and other factors, the levels (as opposed to cyclicality) of 25OHD 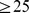 ng/mL may not be generalizable to other countries.

In the general population of the United States, vitamin D levels peak in August and trough in February. In the absence of direct 25OHD measurement or UVB photometry, serum vitamin D levels can be modeled as a function of date, working through a double-log transformation of minimal solar airmass (which is easily calculated from maximal solar altitude, in turn retrieved from an online solar altitude/azimuth table [29]). In the United States, the Oceanic Niño Index is also highly statistically associated with serum 25OHD levels, even net of airmass. It is our hope that the statistical description of serum 25OHD levels in this study will help focus future studies of the role of vitamin D in seasonal infections such as influenza.
